# Functional SNPs of *INCENP* Affect Semen Quality by Alternative Splicing Mode and Binding Affinity with the Target Bta-miR-378 in Chinese Holstein Bulls

**DOI:** 10.1371/journal.pone.0162730

**Published:** 2016-09-26

**Authors:** Juan Liu, Yan Sun, Chunhong Yang, Yan Zhang, Qiang Jiang, Jinming Huang, Zhihua Ju, Xiuge Wang, Jifeng Zhong, Changfa Wang

**Affiliations:** 1 Dairy Cattle Research Center, Shandong Academy of Agricultural Science, Jinan, P. R. China; 2 College of Agronomic Sciences in Shandong Agricultural University, Taian, China; Iowa State University, UNITED STATES

## Abstract

Inner centromere protein (*INCENP*) plays an important role in mitosis and meiosis as the main member of chromosomal passenger protein complex (CPC). To investigate the functional markers of the *INCENP* gene associated with semen quality, the single nucleotide polymorphisms (SNPs) g.19970 A>G and g.34078 T>G were identified and analyzed. The new splice variant *INCENP-TV* is characterized by the deletion of exon 12. The g.19970 A>G in the exonic splicing enhancer (ESE) motif region results in an aberrant splice variant by constructing two minigene expression vectors using the pSPL3 exon capturing vector and transfecting vectors into MLTC-1 cells. *INCENP-TV* was more highly expressed than *INCENP-reference* in adult bull testes. The g.34078 T>G located in the binding region of bta-miR-378 could affect the expression of *INCENP*, which was verified by luciferase assay. To analyze comprehensively the correlation of SNPs with sperm quality, haplotype combinations constructed by g.19970 A>G and g.34078 T>G, as well as g.-692 C>T and g.-556 G>T reported in our previous studies, were analyzed. The bulls with H1H12 and H2H2 exhibited a higher ejaculate volume than those with H2H10 and H9H12, respectively (*P* < *0*.*05*). Bulls with H11H11 and H2H10 exhibited higher initial sperm motility than those with H2H2 (*P < 0*.*05*). The expression levels of *INCENP* in bulls with H1H12 and H11H11 were significantly higher than those in bulls with H9H12 (*P < 0*.*05*), as determined by qRT-PCR. Findings suggest that g.19970 A>G and g.34078 T>G in *INCENP* both of which appear to change the molecular and biological characteristics of the mRNA transcribed from the locus may serve as a biomarkers of male bovine fertility by affecting alternative splicing mode and binding affinity with the target bta-miR-378.

## Introduction

Holstein dairy cows are the species with the highest amount of milk production and the widest breeding range in China. Increasing fertility in dairy cattle is an important goal in a dairy farm. Paternal contributions to the zygote and its development are increasingly recognized as important elements of successful fertilization. Sperm is currently known to provide proteins and RNAs that are critical to subsequent development. The semen quality of bull was not only found to determine the economic benefit of a bull station but it was also reported to significantly affect the reproductive efficiency of cows [1]. Various techniques and protocols can be used to evaluate conventional semen quality parameters, including sperm concentration, motility, progressive motility, and deformity rate, which cannot be easily selected directly because of low heritability [2]. Thus, with the continuous development of molecular genetic techniques, the candidate gene approach in genetics and breeding has been widely popularized in the recent decades [3]. The candidate gene approach could provide theoretical guidance for marker-assisted selection by screening polymorphic loci of candidate genes, analyzing the correlation of polymorphic loci with semen quality, and authenticating molecular markers related to sperm quality. Recent studies have also suggested that a combination of laboratory tests predict fertility [4,5]. Therefore, natural mechanisms, particularly genetic variations, have attracted considerable attention from scientific researchers studying molecular breeding of cattle. Several research teams have identified single nucleotide polymorphism (SNP) sites through a genome-wide association study closely related to semen quality and found that these SNPs affect sperm motility, density, deformity rate, and embryonic development after fertilization [6,7,8].

The potential of spermatogonial stem cells to develop into mature sperm cells require the interaction of multiple genes, with which the cell is originally endowed. Regulated expression of these genes results in efficient functioning throughout the whole cells. Alternative splicing (AS) is a key regulatory mechanism for generating different mature transcripts from the same primary RNA sequence, which also frequently controls output levels and spatiotemporal features of cellular and organismal gene expression programs [9].

MicroRNAs are evolutionarily conserved and short non-coding RNAs (18 to 25 nucleotides) with a function in the regulation of gene expression by mRNA degradation or translational inhibition at the translational level [10]. MiRNAs are post-transcriptional regulators that bind primarily to the 3′-untranslated region (UTR) of the target mRNAs, followed by the coding sequence and 5′-UTR [11]. MicroRNAs are involved in multiple biological phenomena, such as spermatogenesis and early embryogenesis and tumorigenesis [12,13,14]. The SNPs in the binding site of miRNA, namely 3'UTR of the targeted mRNA, have been considered the principal element of microRNA adhesion and are associated with a phenotype, such as semen quality trait [15].

*INCENP* is a versatile domain protein composed of two types of peptides (135 and 155 kD) in close contact with chromosome proteins during cell division. *INCENP*, a part of a protein complex known as the chromosomal passenger complex (CPC), does not only play an important role in cell division [16] but is also closely related to cancer [17,18] and reproductive disorders [19]. Hering et al. (2014) performed a genome-wide association study of high and low sperm motility of Holstein cattle to identify candidate genes associated with semen quality by screening all the genetic distances that significantly mark 1 Mb. They found that *INCENP* located near the rs109416157 significant SNP markers is closely associated with sperm motility and may play an important role in the regulation of sperm quality [20]. Earlier studies also indicated that *INCENP* is mainly involved in cell division, sister chromatid separation, and cytokinesis, thereby causing chromosomal abnormalities and ultimately affecting normal cell division if *INCENP* exhibits abnormal expression [21,22]. Diploid spermatogonia are formed from germline stem cells that undergo mitotic division and differentiation to produce primary spermatocytes [23]. In the process of spermatogenesis, normal chromatid separation is a prerequisite to produce normal sperm [24]. Thus, we predicted that gene polymorphism of *INCENP* may regulate the expression of *INCENP* by affecting cell division during spermatogenesis, thereby regulating sperm quality.

Previous research mainly focused on the role of *INCENP* in cell division, as well as *INCENP* mutations in malignancies, whereas the role of *INCENP* in spermatogenesis was rarely reported. This study focused on the relevance of *INCENP* SNPs with semen quality in Chinese Holstein bulls, as well as possible mechanisms in regulating *INCENP* gene expression. Thus, significant molecular markers associated with the semen quality were identified for theoretical guidance of early cultivation of bulls.

## Materials and Methods

### Animal and tissue samples

A total of 324 normal mature Chinese Holstein bulls from Beijing, Shanghai, and the Shandong bull station were included in the study. Semen samples taken from 100 bulls from the Beijing Dairy Center, 95 bulls from the Shanghai Bright Dairy and Food Co., Ltd., as well as 129 bulls from the Shandong OX Bio-Technology Co., Ltd. were examined. Semen collection and routine veterinary care are carried out by qualified, trained employees. The semen was collected at 3–6 d intervals from each bull using an artificial vagina. Immediately, ejaculate, sperm motility, sperm concentration and the percentage of abnormal sperm were assessed by qualified and trained employees. The fresh semen was then diluted with glycerol–egg yolk–citrate and dispensed into frozen semen straws before cryopreservation. After storage in liquid nitrogen for 5–7 d, two straws were randomly obtained from ejaculate of each bulls and thawed at 38°C for 20 s, and immediately evaluated for the frozen semen quality traits including post-thaw cryopreserved sperm motility according to the guidelines of the World Health Organization [25]. The means and standard errors of sperm quality trait data, including semen volume per ejaculate (mL), sperm motility (%), sperm concentration (×10^8^/mL), post-thaw cryopreserved sperm motility (%), and the percentage of abnormal sperm in the 324 Chinese Holstein bulls are shown in Table 1. Semen samples that met the frozen bovine semen standard (GB/T 4143–2008, China) were collected by professional employees of the respective companies as permitted by the owner. These materials may be applied for SNP screening, genotyping, and association analysis.

**Table 1:** Mean and standard error (SE) of sperm quality traits in 324 Chinese Holstein bulls from 2009 to 2014.

Traits	Mean ± SE
Ejaculate volume (mL)	5.90 ± 0.15
Initial sperm motility (%)	68.03 ± 0.61
Sperm density (×10^8^/mL)	11.07 ± 0.31
Post-thaw cryopreserved sperm motility (%)	42.81 ± 0.58
Deformity rate (%)	16.51 ± 0.31

This study was approved by the Bureau of Animal Husbandry and Veterinary, and the Dairy Cattle Frozen Semen Quality Supervision Testing Center of the Chinese Ministry of Agriculture. The tissue samples, including the heart, liver, spleen, lung, kidney, and testis of adult bulls and calves were collected and immediately snapped and frozen in liquid nitrogen for extraction of RNA isolation and cDNA synthesis. These samples were prepared for the relative expression analysis of the *INCENP* gene from three randomly selected adult Chinese Holstein bulls (3–4 years of age) and three male calves (1–3 days of age), which were killed by halal technique, from the farms of the Dairy Cattle Research Center, Shandong Academy of Agricultural Sciences. Semen samples were collected from 3 Chinese Holstein bulls of each genotype for RNA extraction, respectively.

The murine Leydig tumor cells (MLTC-1) used for analysis of relative luciferase activity and minigene splicing assay were obtained from the Cell Culture Collection of the Chinese Academy of Sciences in Shanghai, China.

### SNP and splice variant

#### Identification of splice variant

Total RNA was extracted using the RNA simple total RNA Kit (Tiangen, Beijing, China) from the testis, heart, liver, spleen, lung, and kidney tissues. RNA amounting to 2 μg was applied to synthesize the first-strand cDNA by using the PrimeScript^TM^ II 1st Strand cDNA Synthesis kit (TaKaRa, DALIAN) stored at -20°C. To search potential splice variants of the bovine *INCENP* gene, the primer pair (S1 Table) was designed to amplify the coding region of the bovine *INCENP* gene. The novel and complete *INCENP* transcripts were distinguished by 2% agarose gel electrophoresis and then purified using the Universal DNA Purification Kit (TIANGEN, Beijing, China). After purification, 1 μL of pEASY-T3 (TransGen Biotech, Beijing, China) was added to 3 μL DNA. The ligation product was transformed into Trans5α (TransGen Biotech, Beijing, China) by heat shocking. Cloning was plated on agar containing 100 mg/mL ampicillin and incubated overnight at 37°C. Picking positive colonies and rapid propagation was then sequenced by Beijing Liuhe Genomics Technology Co., Ltd. The sequencing results were analyzed using DNAMAN and DNAstar. The *INCENP* reference sequence was deposited in GenBank (Accession number: AC_000186.1, http://www.ncbi.nlm.nih.gov/gene/)

#### qRT-PCR of the *INCENP* novel splice variant

To further explore the relative expression of the *INCENP* gross transcript, *INCENP*-reference, and *INCENP*-TV (the novel splice variant) in the testes samples from adult Chinese Holstein bulls and calves, qRT-PCR was conducted using the housekeeping gene *β-actin* as the internal control gene to normalize the data. The qRT-PCR primers that used for amplification of *INCENP* gross transcript (*INCENP-G*), *INCENP-reference*, *INCENP-TV* (the novel splice variant), and *β-actin* are displayed in the S1 Table. In accordance with the instructor’s manual, qRT-PCR was performed using SYBR® Premix Ex Taq^TM^ (TaKaRa, Dalian, China) on a Roche LightCycler 480 machine (Roche Applied Science, Mannheim, Germany). The QRT-PCR reaction system (20 μL) included 10 μL of SYBR® *Premix Ex Taq*^TM^ II, 0.8 μL of forward primer, 0.8 μL of reverse primer (10 μmol/L), 6.4 μL of H_2_O, and 2 μL of cDNA (<200 ng). The qRT-PCR procedure was as follows: 95°C for 30 s followed by 40 cycles of 95°C for 5 s and 60°C for 30 s. A dissociation curve was drawn at 95°C for 5 s, 60°C for 1 min, and 95°C for 15 s. The last stage used for cooling was at 50°C for 30 s. Each sample was run in triplicate. The data were analyzed using the 2^-△△Ct^ method.

#### Screening of splice mutation and PCR-RFLP

A couple of primer pairs synthesized by the Beijing Liuhe Genomics Technology Co., Ltd., were designed using PRIMER PREMIER 5.0 to clone *INCENP* DNA fragments, including SNP1 (g.19970 A>G, rs:109416157) (S1 Table). Sperm DNA was extracted using a high-salt-concentration protocol [26]. The PCR amplification products were sent to Beijing Liuhe Genomics Technology Co., Ltd. for bidirectional sequencing after determining the target band by 1% agarose gel electrophoresis. The sequencing results were analyzed using DNAMAN and ChromasPro software to determine the SNPs of amplified fragment.

According to dCAPs (http://helix.wustl.edu/dcaps/dcaps.html), an appropriate restriction endonuclease was selected for genotyping by PCR-RFLP. Primers (RFLP-1) that clone DNA fragment spanning the SNP1 site (g.19970 A>G, rs: 42658780), restriction endonuclease, and digested fragment size of DNA are shown in S2 Table. The digested product was detected with 2.5% agarose gel electrophoresis. Different genotypes were sorted according to electrophoretic bands.

#### Splicing minigene constructs

Alterations in the binding site of the splicing factor were predicted using ESEfinder 3.0 (http://rulai.cshl.edu/cgi-bin/tools/ESE3/esefinder.cgi). To evaluate alternative splicing in vitro using minigenes, a 716 bp genomic fragment, which spanned the *INCENP* gene 292 bp of intron 11 and 412 bp of intron 12 and entire exon 12, was amplified from the semen genome by a pair of specific primers *INCENP*-pSPL3, (S1 Table) linking the restriction enzyme sites of *EcoR* I and *Xho* I. After restriction, the amplified genomic fragment involving either the wild type or the mutant type of *INCENP* g.19970 A>G, was cloned into the exon trapping vector pSPL3 (Invitrogen, CA, USA). The cloning vectors were then transformed into Trans5a cells plated on agar containing 100 mg/mL ampicillin and incubated overnight at 37°C. Plasmids were isolated using Endo-free Plasmid Mini Kit II (Omega, USA) after they had been verified to contain the correct sequence by direct sequencing. Finally, to avoid mutation during operation, purified plasmids were directly sequenced again before transient transfection assays.

#### Transfection and minigene expression analysis

MLTC-1 cells were cultured in RPMI Medium 1640 (1×) (Gibco® by Life Technologies TM, UK) medium containing 10% fetal bovine serum, penicillin (100 U/mL), and streptomycin (100 μg/mL) at 37°C in a 5% CO_2_ incubator. One day before transfection assay, 0.5 × 10^5^–2 × 10^5^ cells were tiled to a 6-well culture plate to grow to approximately 80% to 90% with an antibiotic free medium. As for *INCENP* minigene transient transfection assays, 4 μg wild-type, mutant-type, as well as empty pSPL3-control plasmid DNA diluted using OPTI-MEM® I Medium (Gibco® by Life Technologies TM, USA), and Lipofectamine 2000 (Invitrogen, Carlsbad, CA, USA) were transfected into MLTC-1 cells together according to the manufacturer’s instructions. The medium was changed with an antibiotic-free medium. MLTC-1 cells were incubated at 37°C in a 5% CO_2_ incubator for 18–48 h prior to testing for transgene expression.

Total RNA was extracted form MLTC-1 cells after 36 h transfection using RNA simple Total RNA Kit (Tiangen, Beijing, China). First-strand cDNA was synthesized using the PrimeScriptTM II 1st Strand cDNA Synthesis Kit (TaKaRa, DALIAN, China) with the pSPL3 vector specific primers SA2 (S1 Table) [19], stored at -20°C. The cDNA was then amplified with pSPL3 vector specific primers SD6 and SA2 (S1 Table) and distinguished by 3% agarose gel electrophoresis. cDNA was finally sequenced and cloned using pEASY-T3. The sequencing results were analyzed using DNAMAN.

### SNP and miRNA

#### Screening of mutation in 3'UTR and PCR-RFLP

On the basis of the DNA sequence of the bovine *INCENP* gene, another primer pair (SNP2) was designed to clone the 3'UTR of *INCENP* (g.34078 T>G, rs: 109416157) and synthesized by the Beijing Genomics Technology Co., Ltd. (S1 Table). SNPs (g.34078 T>G, rs: 42658780) were first identified by bidirectional sequencing. The appropriate restriction endonuclease was selected to genotype SNPs by PCR-RFLP using primer pair of RFLP-2 (S2 Table).

#### Bioinformatics prediction of target miRNA and quantification of miRNA levels

SNPs in 3'UTR, particularly those located in the binding sequence of miRNAs, may play a great role in the regulation of gene expression. To identify miRNAs that may potentially regulate *INCENP* expression, we computationally nominated miRNAs that could contribute to the regulation of *INCENP* expression with g.34378 T>G present in the binding sequence of miRNAs. To ensure the reliability of predictions, multiple prediction algorithms are applied to assess the binding capacity of miRNAs, including RNA22 (https://cm.jefferson.edu/rna22/Interactive/), miRBase (http://www.mirbase.org), and RNAhybrid (http://bibiserv.techfak.uni-bielefeld.de/rnahybrid). Integrating multiple prediction software results, we found the most credible miRNAs as research subjects.

The relative miRNA and bta-miR-378 expressions in testis were determined by quantitative PCR. The detailed steps were described in a previous report [27]. Each miRNA from each sample was analyzed in quadruplicate. Differential miRNA expression was determined using the 2^-△△Ct^ method and shown by a fold change.

#### *INCENP* 3'-UTR plasmid constructs

One pair of specific primers pMIR-3'UTR (S1 Table) that added restriction sites of *Mlu* I and *Hind* III restriction enzymes was designed to clone *INCENP* 3'UTR, which includes g.34378 T>G SNP and contains the assumptive binding sequences for bta-miR-378. The PCR products of g.34378 T>G-*TT* (wild-type) and g.34378 T>G-*GG* (mutant-type) were cloned into PMIR-Report^TM^ Luciferase vector (Ambion). The cloning vectors were then transformed into Trans5a cells to amplify the expression vector of *INCENP* 3'UTR, including the wild-type (pMIR-3'UTR-T) and the mutant-type (pMIR-3'UTR-G). Positive plasmids were isolated using Endo-free Plasmid Mini Kit II (Omega, USA) after they had been verified to contain the correct sequence by direct sequencing.

In addition, microRNA expression vector of bta-miR-378 (precursor sequence agggctcctgactccaggtcctgtgtgttacctcgaaatagcactggacttggagtcagaaggcct) and negative control plasmid miR-control was constructed by the pEZX-MR04 vector according to Wang et al [11]. Bidirectional sequencing was applied to verify the dependability of the related plasmid.

#### Transient transfection assays and analysis of luciferase activity

The MLTC-1 cells were co-transfected with 400 ng of pMIR-3'UTR-T or pMIR-3'UTR-G luciferase reporter plasmid, 50 ng of the internal control pRL-TK plasmid, and 400 ng of each miRNA vector (bta-miR-378 or miR-control) by using OPTI-MEM® I Medium (Invitrogen) and Lipofectamine2000 (Invitrogen) according to the manufacturer’s instructions. The medium was changed with an antibiotic-free medium after 6 h of incubation.

After 36 h of incubation at 37°C in a 5% CO_2_ incubator, MLTC-1 cells were harvested with a passive lysis buffer (Promega), and reporter gene expression was analyzed on the basis of luciferase activity by using the Dual-Luciferase Reporter Assay System (Promega). Luciferase activity was reported in relative light units, and the firefly luciferase activity was normalized against Renilla luciferase activity. All transfections experiments were performed in triplicate and repeated at least thrice in independent experiments.

### Association of *INCENP* gene polymorphisms with semen quality traits

The relationship between the genotype or haplotype combinations of the *INCENP* gene and semen quality traits was analyzed using the least squares method as applied in the general linear models (GLM) procedure in SAS 9.0 (SAS Institute Inc., Cary, NC, USA). The GLM is given as follows [15]:

$$Y_{ijkl}=\mu +H_{i}+P_{j}+S_{\text{k}}+M_{l}+{\text{e}}_{ijkl}$$

where *Y*_*ijkl*_ is the observed value of each semen quality trait, *μ* is the overall mean, *G*_*i*_ is the fixed effect of genotype or haplotype combinations, *P*_*j*_ is the fixed effect of age [j = 2–10; classified as (1) 2 years to 3 years; (2) 4 years to 5 years; (3)≥ 6 years], *S*_*k*_ is the fixed effect of the origin of the bull, *M*_*l*_ is the effect of the farm, and *e*_*ijkl*_ is the random residual error. Values at *P < 0*.*05* and *P < 0*.*01* were regarded as significant.

### qRT-PCR analysis of the mRNA expression

The SNPs of *INCENP* g.19970 A>G, g.34078 T>G, g.-692 C>T and g.-556 G>T [28] were processed to construct haplotype combinations. Trizol Reagent was used for the extraction of sperm RNA consulting Feugang JM [29]. qRT-PCR assay was performed to investigate the differential expression of *INCENP* gross transcript between haplotype combinations by using the SYBR® Premix Ex Taq^TM^ (TaKaRa, Dalian, China) on a Roche LightCycler 480 machine (Roche Applied Science, Mannheim, Germany). Each experiment was performed in triplicate. The relative expression levels of *INCENP* mRNA in different haplotype combinations were normalized by β-actin, the housekeeping internal control gene.

### Statistics

The data for the relative quantification of *INCENP* mRNA and luciferase activity were analyzed using GraphPad Prism 5 and multiple comparisons were performed using Tukey’s test. The results were represented as mean ± SE. The data were analyzed using the 2^-△△Ct^ method to calculate the expression level of *INCENP* gene transcripts. The POPGENE32 software was used to analyze polymorphic information content (*PIC*), heterozygosity (*He*), and effective number of alleles (*Ne*). Genotype frequencies and linkage disequilibrium (LD) was analyzed by SHEsis (http://analysis.bio-x.cn/myAnalysis.php). The general linear model procedure from the statistical analysis software was performed to analyze the correlation between the variants of the *INCENP* gene and semen quality traits. Values at *P < 0*.*05* and P < *0*.*01* were regarded as significant difference and extremely significant difference. Multiple comparisons were performed using Tukey’s test.

## Results

### Identification of one novel bovine *INCENP* splice variant and SNP in intron 11 of *INCENP* and PCR-RFLP

**Fig 1:**
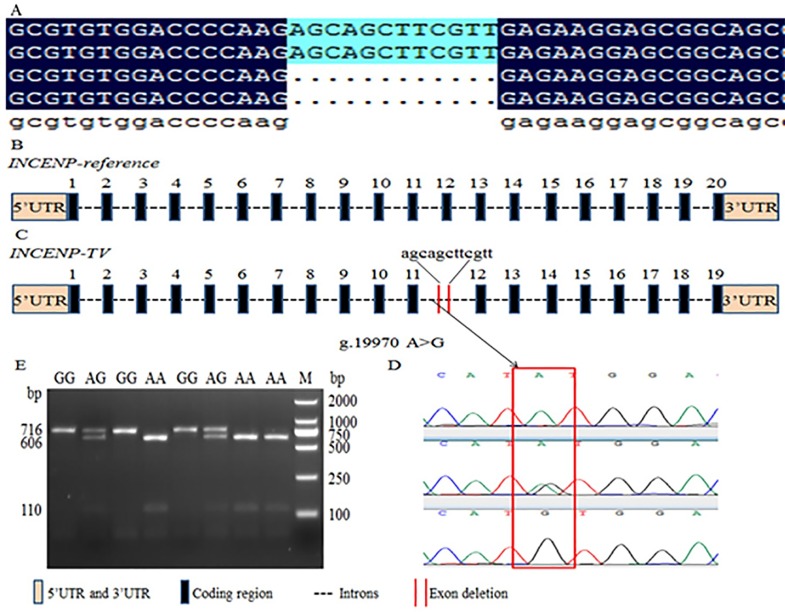
Identification of *INCENP* -TV and SNP g.19970 A>G and PCR-RFLP of of bovine *INCENP* gene. (A) The cloning sequencing result of *INCENP*-cDNA by a pair of primers, F4 and R4. A new transcript *INCENP* -TV which deleted 12 bp was found. (B) Genomic structure of the bovine *INCENP* gene which consists of 20 exons, comprising 2646 bp of coding sequence. (C) The splicing pattern of the *INCENP-TV* splice variant which deletes exon 12, merely composed of 12 bp. (D) The SNP g.19970 A>G (rs:109416157) which lies in the region of intron 11 was discovered by sequencing (E) PCR-RFLP was employed to detect the mutation by *Nde* I. The products of endonuclease digestion were tested in 2.5% agarose gel, showing three genotypes: AA (606 +110 bp), AG (716 + 606 + 110 bp) and GG (716 bp).

Based on specific primers (S1 Table) for *INCENP* cDNA, PCR amplification using various tissue cDNA as a template was performed. These products were purified, cloned, and sequenced (Fig 1A). One novel *INCENP* gene transcript variant (*INCENP-TV*) was found in various tissues referring to transcript of *INCENP* reference (GenBank accession number: XM_002707799.3). The sequence alignment results indicated that the splice variant *INCENP-TV* lacked the 12th exon, which contained 12 bp (Fig 1C). The sequence of *INCENP-TV* was submitted to the National Center of Biotechnology Information (GenBank accession number: KU499879).

To identify the molecular mechanism of the aberrant *INCENP* splice variants, the SNP g.19970 A>G (rs: 42658780), which lies in the region of intron 11, was detected from 60 samples by DNA sequencing and by comparison with the bovine *INCENP* sequence (Fig 1D). Furthermore, in order to clearly understand the situation of genetic polymorphisms in the population of Chinese Holstein bulls, PCR-RFLP was performed to genotype of g.19970 A>G. Digestion of the PCR fragment of *INCENP* g.19970 A>G locus using *Nde* I produced fragments 716 bp long for the genotype GG; 716, 606, and 110 bp for the genotype AG; and 606 and 110 bp for the genotype AA on 2.5% agarose gel (Fig 1E). Statistical data was used for subsequent analysis.

### Expression of the *INCENP* gene in various tissues and quantification of different *INCENP-TV* transcripts in testes of adult bull and calf

**Fig 2:**
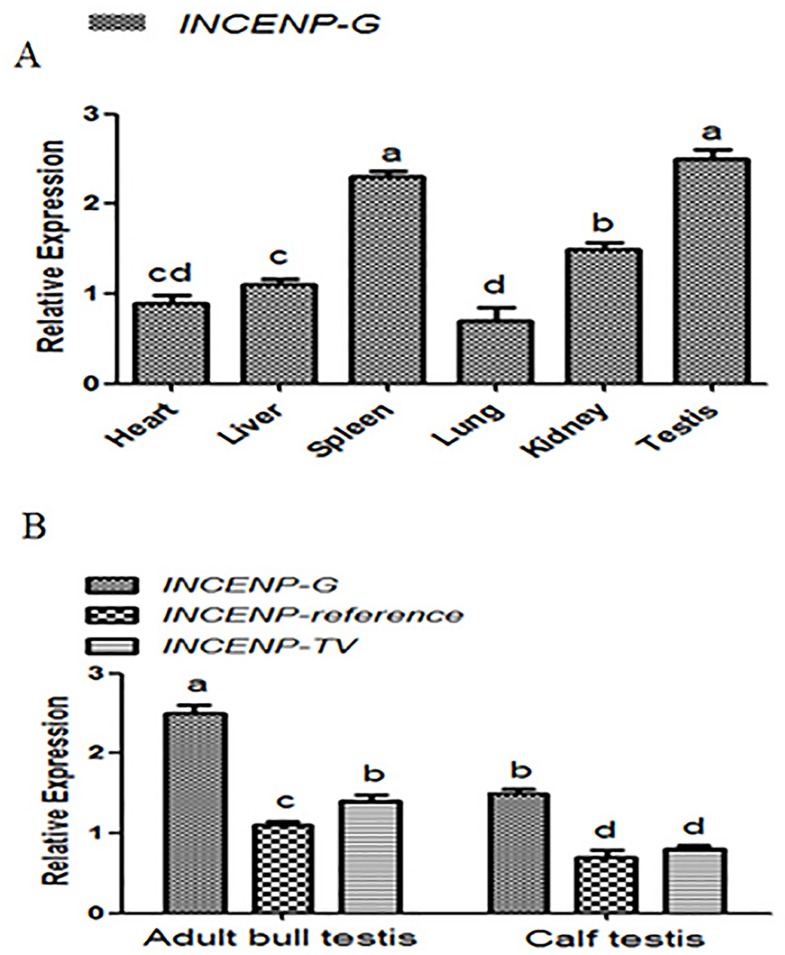
Expression of *INCENP* gene. (A) Expression of the *INCENP* gross transcript (*INCENP* -G) in various tissues in adult bulls using qRT-PCR. (B) Expression of *INCENP* -G, *INCENP*—reference and *INCENP* -TV in testis tissue of adult bulls and calves using qRT-PCR. The vertical bars represent standard errors. Means with different lowercase superscripts above the error bars are significantly different at *P* < 0.05.

To evaluate the mechanism that regulates *INCENP* transcripts, qRT-PCR (quantitative real-time PCR) was conducted to determine the relative expression levels of the *INCENP* gene in the heart, liver, spleen, lung, kidney, and testis, respectively (Fig 2A). The expression of the bovine *INCENP* gene in different tissues exhibited variability. Such expression in spleen and testis was significantly higher than in other adult bull tissues. This result suggested that the *INCENP* gene lacked tissue specificity. We then investigated the mRNA expression of *INCENP-reference* and *INCENP-TV* in adult bull testis, as well as calf testis. The results demonstrated that the expression of *INCENP-reference* and *INCENP-TV* in the adult bull were higher than that in the calf. In addition, *INCENP-TV* was more highly expressed than *INCENP-reference* in the adult bull, whereas *INCENP-TV* seemed to slightly differ from *INCENP-reference* in the calf (Fig 2B).

### Function prediction of SNP in intron 11 of *INCENP*

**Fig 3:**
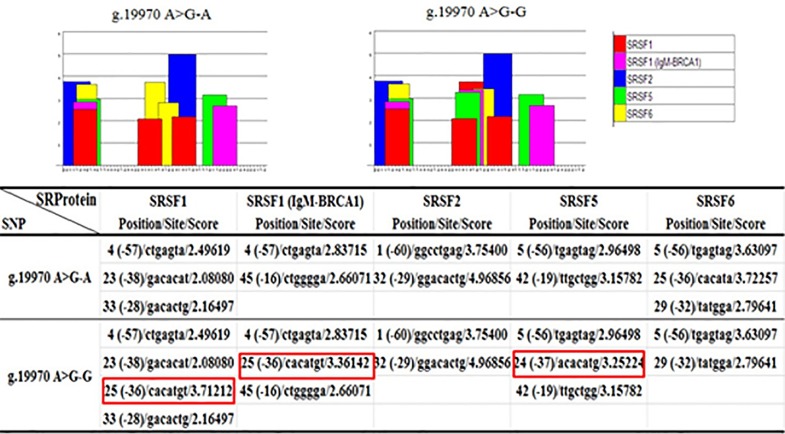
Exonic splice enhancer (ESE) motif threshold scores associated with *INCENP* genotypes. Bar graphs represent scores above the threshold for the ESE motifs within the A or G allele in locus g.19970 A>G. The red square indicates that the introduction of allele G, relative to allele A in locus g.19970 A>G, increased three binding sites of the auxiliary splicing proteins SRSF1, SRSF1 (IgM-BRCA1) and SRSF5 and deleted one of SRSF6.

In view of the discovery and functional understanding of SNP, ESEfinder predicted that the g.19970 A>G located in an exonic splice enhancer (ESE) motif region and mutation added several binding sites for the splicing factors SRSF1, SRSF1 (IgM-BRCA1), SRSF5, and SRSF6 (Fig 3). Thus, we suggest that SNP (g.19970 A>G) may be correlated with the presence or absence of the splice variant *INCENP-TV*. The mutation appears to strengthen the role of the constitutive acceptor splice site, which led to *INCENP-TV* splicing change in mature mRNA during *INCENP* transcription.

### Minigene splicing assay

**Fig 4:**
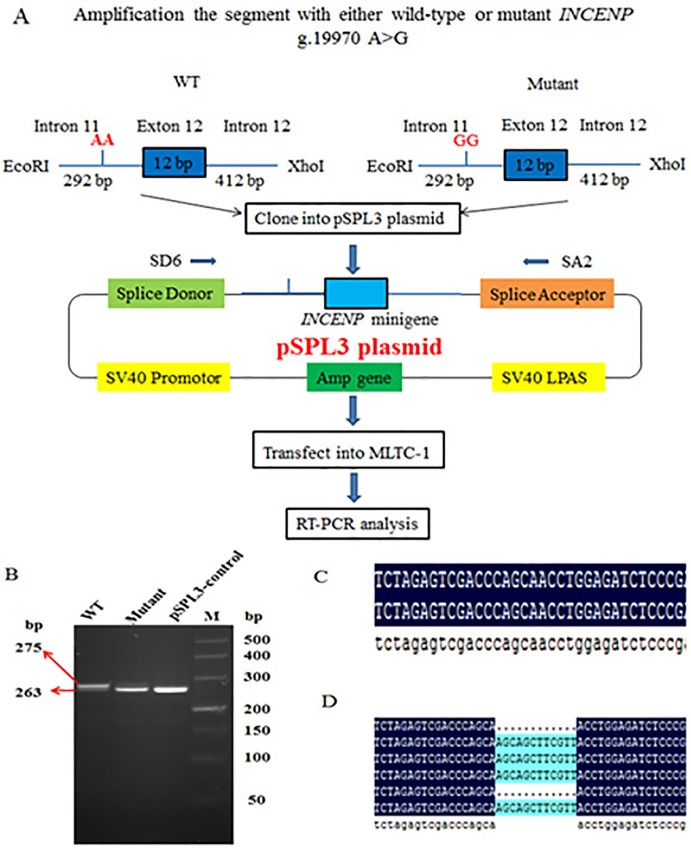
The exon trapping vector pSPL3 used to assay SNP g.19970 A>G function. (A) The pSPL3 vector contains splice donor (SD) and splice acceptor (SA) sites that operate as exons, and a functional intron, with transcription beginning following the SV40 promoter and ending at the LPAS (late poly (A) signal). Wild-type segment and mutant segment containing 292 bp of intron 11, 12 bp of exon 12, 412 bp of intron 12 and harboring either the A or G alleles were separately cloned into the *Eco*RI and *Xho*I cloning sites of the pSPL3 vector. Two minigene expression vectors were transiently transfected into MLTC-1 cells. (B) RT-PCR analysis of the *INCENP* spliced transcripts on 3% agarose gel. Due to the difference of only 12 bp, the result of electrophoresis is not obvious. (C) cDNA was sequenced and the result shown that mutant and empty pSPL3-control expressed a fragment of 263 bp. (D) Cloning sequencing was implemented to verify the fragment of WT. The result showed that WT obtained 275 bp and 263 bp PCR fragment. The sizes of the fragment (275 bp) corresponded to the amplification exon 12 (12 bp) and pSPL3 control plasmid (263 bp). The size of the fragment (263 bp) corresponded to the pSPL3 control plasmid (263 bp).

To determine whether the internal mutation g.19970 A>G in intron 11 leads to an increase in the predicted SR protein motifs and causes diversity of mRNA, the principle of alternative splicing was assessed using minigene splicing assay. We amplified 716 bp genomic fragments, including wild-type g.19970 A>G-A (abbreviated as WT) and mutant g.19970 A>G-G (abbreviated as mutant) into the pSPL3 vector (Fig 4A).

After transiently transfecting the two minigene plasmids and idle pSPL3 into MLTC-1 cells, mRNAs were isolated at 36 h following transfection. The minigene transcripts in these transfected cells were analyzed by RT-PCR using pSPL3 vector-specific primers SD6 and SA2. The result of electrophoresis is not obvious because the difference was only 12 bp (Fig 4B). Thus, cDNA was sequenced, and the results indicated that WT and empty pSPL3-control expressed a fragment of 263 bp (Fig 4C), whereas WT possessed 275 and 263 bp PCR fragments (Fig 4D). Thus, all WT and mutant constructs obtained a 263 bp PCR fragment, and WT constructs also obtained a 275 and 263 bp PCR fragment. Sequencing analysis revealed that one truncated PCR fragment (263 bp) was the same as the empty pSPL3-control (263 bp) sequence because of the exon 12 deficiency, whereas another PCR fragment (275 bp) corresponded to the *INCENP* gene part of the exon 12 (12 bp) and empty pSPL3-control sequence. Minigene splicing assay demonstrated that SNP g.19970 A>G is potentially responsible for the *INCENP-TV* aberrant splicing of 12 bp deletion in exon 12.

### Screening of mutation in 3'UTR and PCR-RFLP

**Fig 5:**
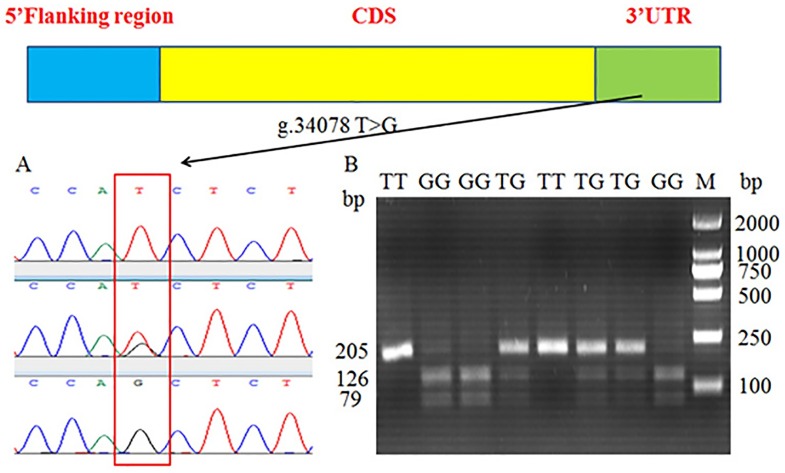
*INCENP* gene structure, location of g.34078 T>G, sequencing results and band patterns of the three genotypes g.34078 T>G. Part A showed Bovine *INCENP* gene structure, location, and results of sequencing of g.34078 T>G. B: 2.5% agarose gel showing band patterns of SNP g.34078 T>G digested with *Alu* I. Digestions of PCR products of *INCENP* g.34078 T>G locus with *Alu* I produced 205-bp bands for homozygous genotype TT, 205-, 126-, and 79-bp bands for heterozygous genotype TG, 126- and 79-bp bands for homozygous genotype GG.

MiRNAs are post-transcriptional regulators that bind primarily to the 3′-untranslated region (UTR) of the target mRNAs. The SNPs in the binding site of miRNA, namely 3'UTR of the targeted mRNA, have been identified as regulatory mechanism of targeted mRNA. In order to verify SNPs in 3'UTR of *INCENP* gene, we detected the polymorphism of 3'UTR. Approximately 637 bp of 3'UTR and partial exon 20 was cloned and screened using a bi-directional DNA sequencing approach to identify whether SNP existed in the 3'UTR of the *INCENP* gene. Only one SNP (g.34078 T>G, rs: 42658780) was found using the *INCENP* gene sequence (GenBank accession number: AC_000186.1) as reference (Fig 5A). Genotyping was then performed by PCR-RFLP with *Alu* I. The products of endonuclease digestion were tested on 2.5% agarose gel, showing three genotypes: TT (205 bp), TG (205 + 126 + 79 bp), and GG (126 +79 bp) (Fig 5B).

**Fig 6:**
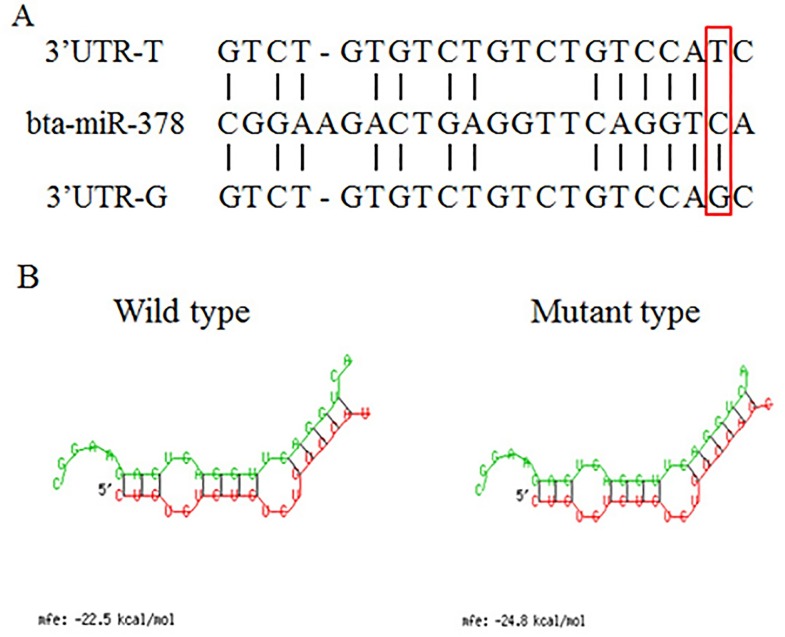
The single-nucleotide polymorphism (g.34078 T>G) located in the seed region of bta-miR-378 binding to the 3′-UTR of the bovine *INCENP* gene. (A) The bta-miR-378: mRNA of *INCENP* interaction is shown. The altered allele is highlighted. (B) Computational modeling of the interaction between miR-378 and 3’-UTR that contains g.34078 T>G-T genotype (wild type) or g.34078 T>G-G (mutant type) was performed on RNAHYBRID software online.

Generally, specificity of the target recognition of miRNAs is crucially dependent on the seed region of the miRNAs regulatory region (nucleotides 2–7 of the 5'-end). Variants, such as SNPs in the miRNA binding site, especially in the seed region, may result in the variation of mRNA or protein levels in organizations. Using RNA22, a method for identifying microRNA binding sites and their corresponding heteroduplexes, we predicted that bta-miR-378 could bind to the 3'UTR of *INCENP* when g.34078 T mutated into G. Furthermore, we predicted the minimum free energy hybridization by RNAhybrid, and the result showed that the minimum free energy of mutant type was lower than wild type, presenting that binding capacity of mutant type and bta-miR-378 was stronger than wild type. All results suggested that g.34078 T>G could influence the association of bta-miR-378 and 3'UTR of *INCENP* (Fig 6). Huang et al. found that bta-miR-378 was highly expressed in the testis by Q-PCR and combined Solexa sequencing with bioinformatics [27].

### Transient transfection assays and analysis of luciferase activity

**Fig 7:**
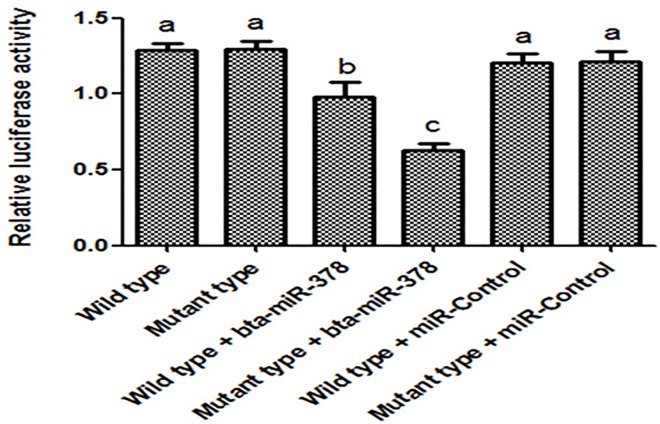
Luciferase activity of different genotypes at g.34078 T>G locus showing PMIR-3′-UTR-T (wild type) or PMIR-3′-UTR-G (mutant type) construct co-transfected with miR-378. Data are from the three transfection experiments with assays performed in six replications. CmiR001-MR04 serves as a scrambled control. Firefly luciferase activity was normalized to the absorbance value of pRL-TK. Vertical bars represent the SE of six replication experiments. Means with different lowercase superscripts above the error bars are significantly different at *P < 0*.*05*.

To verify whether bta-miR-378 binds with the 3'-UTR of *INCENP* or the SNP g.34078 T>G influences differential binding affinity, the luciferase reporter assay was implemented. The results revealed that the bta-miR-378 reduced the luciferase reporter gene activity, indicating that bta-miR-378 suppressed expression of reporter gene. This seems to be different from forecasting results of RNA22. Nevertheless, the data also showed that expression of reporter gene of mutant type 3'-UTR was below wild type (P < 0.05), suggesting a higher binding affinity for the mutant type 3'-UTR compared with the wild-type 3'-UTR, which is consistent with the computational prediction results (Fig 7).

### Association of *INCENP* gene polymorphisms with semen quality traits

Relative genetic parameters of the bovine *INCENP* gene were statistically analyzed (S3 Table). The results demonstrated that *AG* genotypes predominate in the population, and A allele frequencies are greater than G for g.19970 A>G. Meanwhile, TG genotypes predominate in the population, and G allele frequencies are greater than T for g.34078 T>G. The results of PIC, He, Ne, and χ^2^ tests showed that P values of g.19970 A>G and g.34078 T>G were greater than 0.05, indicating that the genotype and gene frequencies of the two mutations reached the Hardy–Weinberg equilibrium state, and polymorphic sites were moderate polymorphisms in the population.

We then analyzed the effects of the two genetic variations on semen quality traits by using the SAS program (Table 2). Multiple comparisons were performed using Tukey’s test. We found that the initial sperm motility of bulls with g.19970 A>G-GG genotype was greater than that with AA genotype (*P<0*.*05*). Meanwhile, ejaculate volume, sperm density, post-thaw cryopreserved sperm motility, and the percentage of abnormal spermatozoa between different genotypes had no significant difference (*P> 0*.*05*). Likewise, the initial sperm motility of bulls with the g.34078 T>G-TT genotype was greater than that with AA genotype (*P<0*.*05*). Well known haplotype combinations are considered better than a single SNP for assessing semen quality. Thus, we constructed haplotype combinations by g.19970 A>G and g.34078 T>G, as well as g.-692 C>T and g.-556 G>T reported in our previous study [28]. Linkage equilibrium analysis indicated that the frequency of simultaneous heredity between those 4 SNPs was significantly higher than the expected random frequencies, thereby suggesting a strong linkage disequilibrium (LD) (S1A Fig).

**Table 2:** Least-square means and standard error of semen quality traits of different genotypes in the *INCENP* gene of Chinese Holstein bulls.

SNP Loci	Genotype	Ejaculate volume (mL)	Sperm density (×108/mL)	Initial sperm Motility (%)	Post-thaw cryopreserved sperm motility (%)	Deformity Rate (%)
g.19970 A>G	AA	5.99± 0.22	11.19 ± 0.42	66.98 ± 0.83^b^	42.28 ± 0.80	16.52 ± 0.50
	AG	5.89 ± 0.18	10.80 ± 0.34	68.11 ± 0.67	43.26 ± 0.64	16.51 ± 0.42
	GG	5.80 ± 0.31	11.68± 0.59	69.82 ± 1.17^a^	42.53± 1.12	16.47± 0.72
g.34078 T>G	TT	5.64 ± 0.30	11.65 ± 0.58	69.70 ± 1.15^a^	42.45± 1.10	16.32 ± 0.70
	TG	5.97 ± 0.18	10.94 ± 0.35	68.29 ± 0.69	43.23 ± 0.66	16.71 ±0.42
	GG	5.94 ± 0.21	10.97 ± 0.41	66.83 ± 0.81^b^	42.42 ± 0.78	16.33 ± 0.50

Note: For each one SNP, means with different lowercase letters within the same column are significantly different (*P < 0*.*05*). Means without lowercase letter and means with different lowercase letters are not significant difference (*P > 0*.*05*).

Four SNPs should be 12 haplotypes, as follows: H1 (CGAT), H2 (CGAG), H3 (CGGT), H4 (CGGG), H5 (TGAT), H6 (TGAG), H7 (TGGT), H8 (TGGG), H9 (TTAT), H10 (TTAG), H11 (TTGT) and H12 (TTGG). The first position refers to the g.-556 SNP, the second position refers to the g.-692 SNP, the third position refers to the g.19970 SNP and the fourth position refers to the g.34708 SNP. However, only 7 haplotypes—H1, H2, H3, H9, H10, H11 and H12—were identified with the following frequencies: 20.37%, 29.01%, 0.62%, 4.63%, 4.32%, 16.05%, and 25%, respectively, in the test group (S1B Fig). Similarly, 13 haplotype combinations were discovered, as follows: H10H10, H10H12, H11H11, H1H10, H1H11, H1H12, H1H2, H1H3, H2H10, H2H12, H2H2, H3H11 and H9H12. The number of bulls with H10H10, H1H11, H1H3, and H3H11 was no more than 4. Thus, we processed these data via correlation analysis. Correlation analysis indicated that the ejaculate volume of bulls with H1H12 and H2H2 was significantly greater than that of bulls with H2H10 and H11H11 (*P<0*.*05*). Furthermore, the initial sperm motility of bulls with H11H11 and H2H10 was greater than that of bulls with H2H2 (*P<0*.*05*) (Table 3).

**Table 3:** Least-square mean and standard error of semen quality traits of different *INCENP* haplotype combinations in Chinese Holstein bulls.

Genotypes combination/Sample number	Ejaculate volume (mL)	Sperm density (×108/mL)	Initial sperm Motility(%)	Post-thaw cryopreserved sperm motility (%)	Deformity Rate (%)
H10H12/4	5.54±1.12	8.88±2.23	66.31±4.32	40.42±4.18	19.08±3.23
H11H11/50	5.70±0.32	11.73±0.63	69.84±1.22^a^	42.65±1.18	16.47±0.78
H1H10/4	6.32±1.12	11.58±2.23	66.60±4.32	45.63±4.18	18.10±2.28
H1H12/120	6.19±0.20^a^	11.15±0.41	68.71±0.79	43.25±0.76	16.38±0.49
H1H2/4	6.05±1.12	12.14±2.23	67.29±4.32	40.47±4.18	18.95±2.28
H2H10/16	4.89±0.56^b^	10.90±1.11	71.23±2.16^a^	45.27±2.09	16.12±1.61
H2H12/8	5.56±0.79	9.82±1.57	65.28±3.05	45.31±2.95	16.71±2.28
H2H2/80	6.21±0.25^a^	11.19±0.50	66.34±0.96^b^	41.66±0.93	16.32±0.57
H9H12/30	5.11±0.41^b^	9.90±0.81	66.83±1.58	43.32±1.53	17.16±1.08

Note: H1 = CGAT; H2 = CGAG; H3 = CGGT; H9 = TTAT; H10 = TTAG; H11 = TTGT; H12 = TTGG; Means with different lowercase letters within the same column are significantly different (*P < 0*.*05*). Means without lowercase letter and means with different lowercase letters within the same column are not significant difference (*P > 0*.*05*).

### mRNA expression of haplotype combination of SNPs

**Fig 8:**
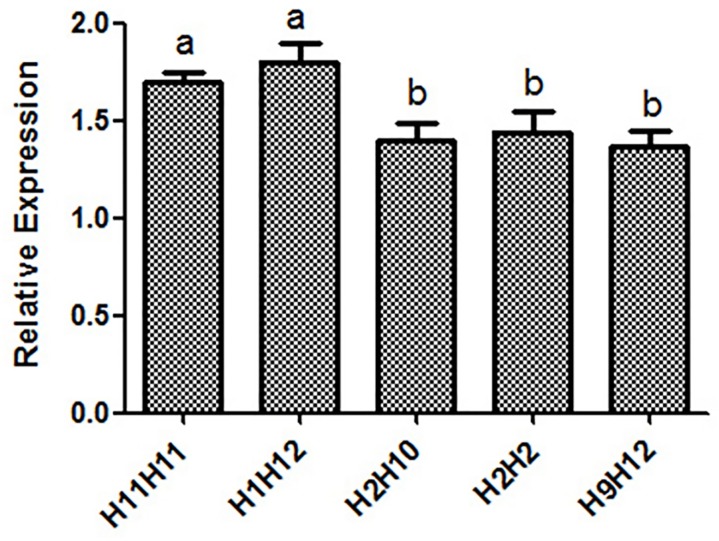
Expression of haplotype combination of *INCENP* gross transcript *(INCENP -G)*. The *INCENP* with the haplotype combinations H11H11 and H1H12 showed a significantly (*P < 0*.*05*) higher mRNA expression compared that with the haplotype combinations H2H10, H2H2 and H9H12. H1 = CGAT; H2 = CGAG; H9 = TTAT; H10 = TTAG; H11 = TTGT; H12 = TTGG. Means with different lowercase superscripts above the error bars are significantly different at *P < 0*.*05*.

Relative quantification was performed to illustrate the relative mRNA expression levels of *INCENP* gross transcript (*INCENP-G*) in the sperm cells from different haplotype combinations of bulls. The *INCENP* with the mutant haplotype H11H11 and H1H12 showed a significantly (*P*<0.05) higher mRNA expression compared with the haplotype combinations H2H10, H2H2, and H9H12 (Fig 8). Therefore, the SNPs, g.19970 A>G, g.34078 T>G, g.-692 C>T, and g.-556 G>T can potentially influence the mRNA expression of *INCENP*.

## Discussion

Changes in gene components may affect gene expression and alter gene functional activity. SNPs are the most common way of gene mutation, mainly referring to a single base mutation in the DNA sequence, including conversions, transversions, insertions, and deletions. Coding region SNPs (cSNPs) may have an important significance in the structure and function of the protein and the study of hereditary diseases [30]. SNPs in the 5'flanking region, especially transcription factor binding sites, may regulate gene expression by affecting the promoter activity [31]. SNPs in the 3'UTR, especially binding sites of microRNA, regulate gene expression at either transcriptional or post-transcriptional level [32]. In our previous research, two polymorphisms—g.-556 G> T and g.-692 C> T—in the 5' flanking region of *INCENP* were examined using PCR-RFLP [33] in Chinese Holstein bull; g. -556 G> T was closely related to initial sperm motility, thereby suggesting that SNPs influence *INCENP* gene expression by regulating promoter activity [28]. To further investigate the role of *INCENP* in influencing semen trait, two other SNPs (g.19970 A>G in intron 11 and g.34078 T>G in 3'UTR) were identified and genotyped in *INCENP* by sequencing and PCR-RFLP. Association analysis indicated that SNPs g.19970 A>G and g.34078 T>G were significantly correlated with the initial sperm motility of Chinese Holstein bulls if the environment and peculiarities in the AI stations were disregarded. Our results coincided with the results of genome-wide association in the study by Hering et al. (2014) that *INCENP* located near rs109416157 significant SNP markers was closely associated with sperm motility [20]. Fertilization is regarded as a test of sperm physical strength and endurance. Although one ejaculation can produce billions of sperm, the vast majority of sperm die because of loss of vitality before reaching the ampulla of the fallopian tube; in addition, only the fastest sperm that successfully reaches the correct fertilization site can complete fertilization with eggs. Therefore, sperm motility is a key indicator of evaluating sperm quality [34], and a study on the correlation of *INCENP* gene SNPs with Chinese Holstein bull semen traits has great significance in guiding Chinese Holstein cattle breeding.

Compared with exploring the correlation of SNPs in a gene with biological traits, research on haplotype combinations between multiple SNPs may be more constructive, among which interaction among SNPs can be considered [35]. Furthermore, the results of linkage equilibrium analysis indicated that the frequency of simultaneous heredity between those SNPs (g.-556 G>T, g.-692 C>T, g.19970 A>G and g.34078 T>G) was significantly higher than the expected random frequencies, thereby suggesting a strong linkage disequilibrium (LD). Synthesizing the four SNPs (g.-556 G>T, g.-692 C>T, g.19970 A>G and g.34078 T>G), haplotype combinations was performed to construct and analyze. According to results of association analysis and qRT-PCR, bulls with H11H11 exhibited higher initial sperm motility and mRNA expression, and bulls with H1H12 had higher ejaculate volume and higher mRNA expression, and bulls with H9H12 had the lower ejaculate volume and the lower mRNA expression. Thus, the SNPs g.-556 G>T, g.-692 C>T, g.19970 A>G, and g.34078 T>G of *INCENP* may be responsible for the differences in the ejaculate volume and initial sperm motility of bull sperm. Our findings suggested that these four SNPs contribute semen traits by influencing *INCENP* gene expression. Consistent with these findings, other studies have revealed relevant supporting information. *INCENP* is a short attachment protein, changing expression sites during cell division [36]. In the mid-term of cell division, *INCENP* is located in the centromere, which connects sister chromatids. This occurrence suggests that *INCENP* is related to the separation of sister chromatids and plays a crucial role in cell division. In late-term cell division, considering that cell division is an important stage of spermatogenesis, the absence of *INCENP* can lead to dysregulation of spermatogenesis, thereby resulting in chromosomal aberration and cytokinesis. Our study further demonstrated the potentially important role of INCENP in spermatogenesis and regulation of semen quality. Interestingly, the bulls with H2H10 had lower ejaculate volume and higher initial sperm motility, and those with H2H2 had higher ejaculate volume and the lower initial sperm motility. However, both the bulls with H2H2 and H2H10 exhibit lower mRNA expression. These results suggest that the biological functions of complex molecular mechanisms of *INCENP* exist, as well as gene expression regulation at the transcriptional and post-transcriptional levels.

Alternative splicing (AS) is a basic and important mechanism for regulating gene expression patterns in different tissues and disease states by generating multiple mRNAs from the same gene transcript [37]. In our research, a new *INCENP* transcript variant named *INCENP-TV* was identified following the cloning of *INCENP* mRNA. Alternative splicing in spermatogenic cells is critical for normal spermatogenesis and male fertility [38]. Recent studies have reported that survival motor neuron (SMN) protein, has recently been implicated in spermatogenesis by an adult-specific splicing switch [39]. We suggest that *INCENP* transcript variant is involved in spermatogenesis, therefore, qRT-PCR was performed to estimate *INCENP* expression. The results showed that the expression of bovine *INCENP* gene in different tissues exhibits variability. This expression in spleen and testis was significantly higher than that in other tissues in adult bull tissues, thereby suggesting that *INCENP* gene had no tissue specificity. This finding seemed to correspond to the important role of *INCENP* in cell division because various tissues were under continuous cell replacement although cell division between them varies. Compared with the *INCENP-reference*, the exon 12 that was composed of 12 bp was deleted in *INCENP-TV*. Although *INCENP* was found in various tissues, a significant difference was observed between the expression levels of *INCENP-reference* and *INCENP-TV*. The results demonstrated that not only *INCENP-reference* but also *INCENP-TV* was more highly expressed in adult bull than in calf. In addition, *INCENP-TV* was more highly expressed than *INCENP-reference* in adult bull, whereas *INCENP-TV* seemed to slightly differ from *INCENP-referenc*e in calf. Spermatogenesis mainly occurs in seminiferous tubules of sexually mature male testicles. The difference in expression between the adult bull and the calf suggested that *INCENP* gene was associated with the generation of male reproductive cells.

Previous studies have found that SNPs located in exon splicing enhancer (ESE) are among the main causes of abnormal splicing [40]. For example, an intronic SNP rs35599367 (CYP3A4*22, located in intron 6) affected the intron 6 retention in liver-derived HepG2 cells [41]. These finding suggested that SNPs exhibited a strong connection with alternative splicing and correlative phenotypic characters. Thus, we first used an ESEfinder bioinformatics tool to predict whether the g.19970 A>G in intron 11 affected the binding capacity with splicing proteins. The prediction results showed that g.19970 A>G in intron 11 was located within an exonic splice enhancer (ESE) motif. The three SNP-derived potential SR protein binding sites for the splicing factors, namely, SRSF1, SRSF (IgM-BRCA1), and SRSF5 were increased while binding sites for SRSF6 was removed. Splicing regulation is involved in multiple splicing factors. Among many factors, the SRSF proteins (serine/arginine-rich splicing factor, SRSF) are essentially involved in nearly every step of the process and exhibit diverse functions, ranging from their role in constitutive and alternative splicing to virtually all aspects of mRNA metabolism [42,43]. SRSF proteins are a family of highly conserved cellular splicing factors and major modulators of alternative splicing; in addition, they are characterized by the presence of an RNA-recognition motif and Ser/Arg (SR) dipeptides [44]. We conjectured that the increase in the three splicing factors may affect *INCENP* splice variant splicing. To further evaluate the contribution of the g.19970 A>G in the regulation of *INCENP-TV*, we constructed two minigene expression vectors transiently transfected into MLTC-1 cells, as analyzed by RT-PCR. The results indicated that the mutant-type GG construct permitted the removal of exon 12, whereas the wild-type AA construct permitted two types of transcription. This experiment was consistent with the aforementioned prediction results. Thus, we suggested that the SNP g.19970 A>G contributes to the aberrant splicing of *INCENP-TV*. Palhais et al. (2015) reported that an intronic mutation (c.903+469T>C) in the 5-methyltetrahydrofolate-homocysteine methyltransferase reductase or MTRR gene strengthened an SRSF1 binding site in an ESE, thereby leading to intron retention [45]. The exon 29 c.3535A>T in the alpha-2-macroglobulin (*A2M*) gene reportedly increased two binding sites for the splicing factors SRSF2 and SRSF5, which resulted in the A2M-AS4 aberrant splice variant [40].

The mature miRNAs bind to their complementary mRNA sites, namely the 3' untranslated region (3'UTR) of the mRNA of target genes, mediated mRNA degradation, and post-transcriptional repression. The genetic variants in the 3'-UTR may affect bovine semen quality traits and expression levels by translational suppression/activation in which SNPs alter the binding of the miRNA to the 3'-UTR of the target gene [46]. In the current study, we found that the bovine *INCENP* gene is a target for bta-miR-378 and the SNP g.34078 T>G located at the seed sites of bta-miR-378. Generally, specificity of the target recognition of miRNAs is crucially dependent on the seed region of the miRNAs regulatory region (nucleotides 2–7 of the 5'-end) [47]. Variants, such as SNPs in the miRNA binding site, especially in the seed region, may result in the variation of mRNA or protein levels in organizations. Mencía et al. identified two mutations in the seed region of miR96 in two Spanish families, thereby resulting in autosomal-dominant and progressive hearing loss [48]. Further transfection experiment in vitro was performed. The SNP-altered binding affinity of bta-miR-378 had a higher binding affinity for the mutant type 3'-UTR compared with the wild type 3'-UTR. This result indicated that the SNP g.34078 T>G altered the expression of *INCENP* mRNA by bta-miR-378. Metzler-Guillemain C et al [49] indicated that alterations of microRNAs may contribute to mRNA expression in spermatogenic cells, thereby it plays great significance on to discuss essential role of miRNAs on spermatogenesis.

In conclusion, g.19970 A>G and g.34078 T>G were functional SNPs in *INCENP*, which are possibly implicated in spermatogenesis and normal morphological characteristics of Chinese Holstein bull sperm by alternative splicing and affect the binding capacity to bta-miR-378. We proposed that the SNPs of *INCENP* may be used to successfully select semen quality traits in Chinese Holstein bulls in the dairy industry. Furthermore, these findings could help us appreciate the complex molecular mechanisms of *INCENP* biological functions and gene expression regulation at the transcriptional and post-transcriptional level.

## Supporting Information

S1 Fig Linkage disequilibrium (LD) tests for four SNPs, haplotypes and combination of haplotypes in the *INCENP* gene. A: Blank box represents the region from the translation start codon (ATG) to the stop codon. Green box represents *INCENP* 5'-flanking region. Blue box represents the 3'-non-coding untranslated region (UTR) of *INCENP*. The icons on the left panel represent D'. LD relationship between each two SNPs was analyzed by SHESIS software. The D' value for the comparison of the two SNPs is shown in black numbers. B: H1 (CGAT), H2 (CGAG), H3 (CGGT), H9 (TTAT), H10 (TTAG), H11 (TTGT) and H12 (TTGG).
(TIF)

S1 Table Primers used for bovine *INCENP* cDNA amplification, screening SNPs, relative expression of the *INCENP* gene and constructs of plasmid. Underlined letters refer to protective bases and restriction endonuclease sites.
(DOCX)

S2 Table PCR-RFLP tests for bovine *INCENP* gene genotyping. (DOCX)

S3 Table Genotypic, allelic frequencies, and other genetic indices (*He*, *PIC*, *Ne*, *P*) of the *INCENP* gene at positions: g.19970 A>G, g.34078 T>G. *He*, heterozygosities; *Ne*, effective of alleles; *PIC*, polymorphism information content.
(DOCX)
