# Supplementary figures and images for: Functional SNPs of INCENP Affect Semen Quality by Alternative Splicing Mode and Binding Affinity with the Target Bta-miR-378 in Chinese Holstein Bulls
Source: PLoS One. 2016 Sep 26;11(9):e0162730. doi: 10.1371/journal.pone.0162730 (PMC5036895; doi:10.1371/journal.pone.0162730)

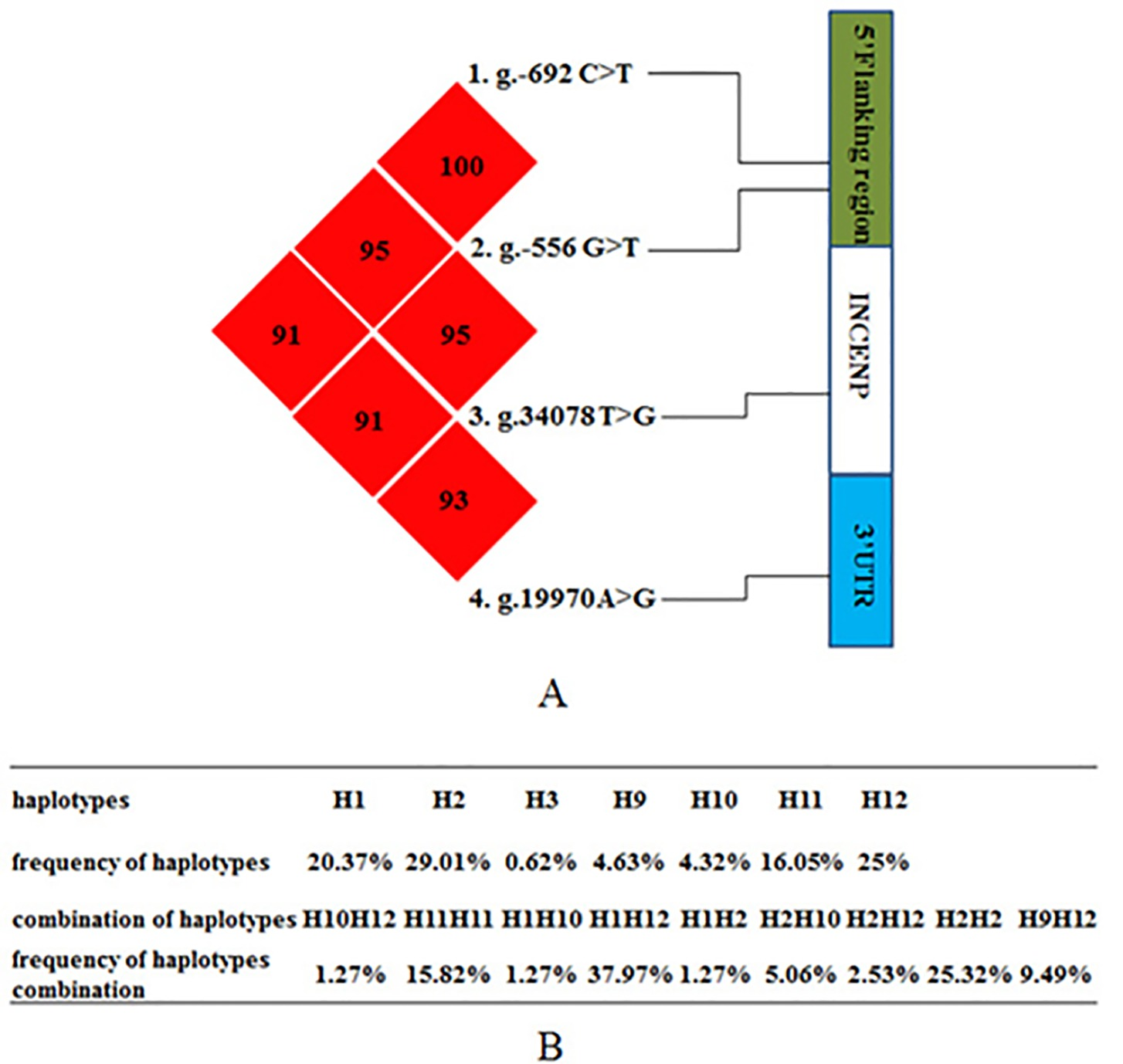

Supplement: S1 Fig — A: Blank box represents the region from the translation start codon (ATG) to the stop codon. Green box represents INCENP 5'-flanking region. Blue box represents the 3'-non-coding untranslated region (UTR) of INCENP. The icons on the left panel represent D'. LD relationship between each two SNPs was analyzed by SHESIS software. The D' value for the comparison of the two SNPs is shown in black numbers. B: H1 (CGAT), H2 (CGAG), H3 (CGGT), H9 (TTAT), H10 (TTAG), H11 (TTGT) and H12 (TTGG). (TIF) [file pone.0162730.s001.tif]
