# Supplementary material for: Functional SNPs of INCENP Affect Semen Quality by Alternative Splicing Mode and Binding Affinity with the Target Bta-miR-378 in Chinese Holstein Bulls
Source: PLoS One. 2016 Sep 26;11(9):e0162730. doi: 10.1371/journal.pone.0162730 (PMC5036895; doi:10.1371/journal.pone.0162730)
Supplement: S1 Table — Underlined letters refer to protective bases and restriction endonuclease sites. (DOCX) [file pone.0162730.s002.docx]

**S1 Table Primers used for bovine *INCENP* cDNA amplification, screening SNPs, relative expression of the *INCENP* gene and constructs of plasmid**

| **Primers** | **Primer sequence (5'-3')** | **PCR product size (bp)** | **Annealing temperature (°C)** |
| --- | --- | --- | --- |
| cDNA | F1:CTTAGAGTTTTGCAGCCGCC | 710 | 61 |
|  | R1:GTTGGGCTCTCCTTGGGATT |  |  |
|  | F2:CAGGTCTCCCACTGTCCTGA | 234 | 55 |
|  | R2:GTGACAGACTCCAGCCTTCC |  |  |
|  | F3:TTCAACACCCCAGAAGGCAG | 705 | 59 |
|  | R3:TCATTCACCGCCTGCTTGTA |  |  |
|  | F4:AACATCACCCTTAGCCCACC | 1083 or 1071 | 59 |
|  | R4:CACGTCCACGGTCACATTCA |  |  |
|  | F5:CCTCCGCAAGGTGCTACA | 1080 | 59 |
|  | R5:TGCTGTCCACAGACCAGGTG |  |  |
| g.19970 A>G (SNP1) | F:TCATCAAGCGCAACACTCCT | 621 | 59 |
|  | R:CCCTTAACGCTCCCACCAAT |  |  |
| g.34078 T>G (SNP2) | F:GCCGTCATCCGCCAGTATTA | 637 | 59 |
|  | R:TTCTCAGGGTTGTTCAGGGC |  |  |
| *INCENP-G* | F:GCAATAGCCTGAATGTGACCG | 126 | 60 |
|  | R:CACTGTTTAGATCCATCCCGTAG |  |  |
| *INCENP-reference* | F:CCACCCTGTCCTTCCAGCAA | 164 | 60 |
|  | R:AAGCTGCTCTTGGGGTCCA |  |  |
| *INCENP-TV* | F:GGAGAAGGAGCGGCAGCG | 174 | 60 |
|  | R:TCCTTCATCTGCTCCACCC |  |  |
| *β-actin* | F:GCACAATGAAGATCAAGATCATC | 173 | 60 |
|  | R:CTAACAGTCCGCCTAGAAGCA |  |  |
| *INCENP-*  pSPL3 | F:CGGAATTCGGGCCGGGAAGTTACAGAAC | 716 | 59 |
|  | R:CCGCTCGAGCAAGGCAGCAGCTTAGGTCT |  |  |
| pSPL3 | SD6:TCTGAGTCACCTGGACAACC | 275 or 263 | 59 |
|  | SA2:ATCTCAGTGGTATTTGTGAGC |  |  |
| pMIR-3'UTR | F:CGACGCGTGCCGTCATCCGCCAGTATTA | 351 | 60 |
|  | R:CCCAAGCTTGCCACTCTTCACCCATGACA |  |  |

Note: Underlined letters refer to protective bases and restriction endonuclease sites.
