# Supplementary material for: Functional SNPs of INCENP Affect Semen Quality by Alternative Splicing Mode and Binding Affinity with the Target Bta-miR-378 in Chinese Holstein Bulls
Source: PLoS One. 2016 Sep 26;11(9):e0162730. doi: 10.1371/journal.pone.0162730 (PMC5036895; doi:10.1371/journal.pone.0162730)
Supplement: S2 Table — (DOCX) [file pone.0162730.s003.docx]

**S2 Table PCR-RFLP tests for bovine *INCENP* gene genotyping**

| **Primers** | **Primer sequence (5'-3')** | **Restriction endonuclease** | **Fragment size (bp)** |
| --- | --- | --- | --- |
|  | F:GGGCCGGGAAGTTACAGAAC |  | AA:606, 110 |
| RFLP-1 |  | *Nde* I | AG:716, 606,110 |
|  | R:CAAGGCAGCAGCTTAGGTCT |  | GG:716 |
|  | F:GCCTACAGCCTGAAGAAG |  | TT:205 |
| RFLP-2 |  | *Alu* I | TG:205, 126, 79 |
|  | R:AGTGCTGTCCACAGACCA |  | GG:126,79 |
