# Supplementary material for: Functional SNPs of INCENP Affect Semen Quality by Alternative Splicing Mode and Binding Affinity with the Target Bta-miR-378 in Chinese Holstein Bulls
Source: PLoS One. 2016 Sep 26;11(9):e0162730. doi: 10.1371/journal.pone.0162730 (PMC5036895; doi:10.1371/journal.pone.0162730)
Supplement: S3 Table — He, heterozygosities; Ne, effective of alleles; PIC, polymorphism information content. (DOCX) [file pone.0162730.s004.docx]

**S3 Table Genotypic, allelic frequencies, and other genetic indices (He, PIC, Ne, *P*) of the *INCENP* gene at positions: g.19970 A>G, g.34078 T>G**

| **SNP Loci** | **Genotype** | **Sample number** | **Genotypic frequencies** | **Allelic frequencies** | **χ^2^ (*P*value)** | ***PIC*** | ***He*** | ***Ne*** |
| --- | --- | --- | --- | --- | --- | --- | --- | --- |
| g.19970 A>G | AA | 106 | 0.327 | A:0.58 | 0.248 (0.619) |  |  |  |
|  | AG | 164 | 0.506 |  |  | 0.368 | 0.487 | 1.95 |
|  | GG | 54 | 0.167 | G:0.42 |  |  |  |  |
| g.34078 T>G | TT | 56 | 0.173 | T:0.414 | 0.018 (0.894) |  |  |  |
|  | TG | 156 | 0.481 |  |  | 0.367 | 0.485 | 1.942 |
|  | GG | 112 | 0.346 | G:0.586 |  |  |  |  |

Note: *He*, heterozygosities; *Ne*, effective of alleles; *PIC*, polymorphism information content.
